# Gait analysis reveals new outcome measures for monitoring disease progression in individuals with late-onset Pompe disease

**DOI:** 10.1186/s12984-026-01898-8

**Published:** 2026-03-09

**Authors:** Mireia Claramunt-Molet, Jordi Pegueroles, Ariadna Pi-Cervera, Mari Rico, Sebastian Idelsohn-Zielonka, Cristina Domínguez-González, Manuela Corti, Virgilia Antón, Stephanie M. Salabarria, Karen Wong, Meredith K. James, Barry J. Byrne, Jordi Díaz-Manera

**Affiliations:** 1 Ephion Health, Barcelona, 08005 Spain; 2 Unit of Digital Health, Centre Tecnològic de Catalunya, Eurecat, Barcelona, Spain; 3 Neuromuscular disorders Unit, Neurology department, 12 de Octubre Hospital, Madrid, Spain; 4 imas12 Research Institute, Madrid, Spain; 5 Powell Gene Therapy Center, University of Florida, Gainesville, USA; 6 Universidad de Castilla La Mancha, Toledo, Spain; 7 The John Walton Muscular Dystrophy Research Centre, Newcastle University Translational and Clinical Research Institute, Newcastle Upon Tyne, UK; 8 Neuromuscular diseases laboratory, Hospital Sant Pau Research Institute, Barcelona, Spain; 9 Centro de Investigación Biomédica en Red en Enfermedades Raras (CIBERER), Barcelona, Spain

**Keywords:** Late-onset Pompe disease, Wearable gait analysis, Disease progression, Biomechanical biomarkers, Neuromuscular disorders

## Abstract

**Background:**

Late-onset Pompe disease (LOPD) presents with progressive muscle weakness, often leading to functional impairment that is challenging to monitor with conventional assessments. This study aims to develop and validate novel gait-based outcome measures for monitoring disease progression in individuals with LOPD.

**Methods:**

Longitudinal study with genetically confirmed LOPD patients and age-and gait velocity-matched healthy controls that were assessed over a two-year period using the Ephion Mobility system, which integrates inertial sensors, plantar pressure insoles, and surface electromyography. All participants completed a free walking test (10–15 m at self-selected pace) and the 6-minute walk test (6MWT). Differences in gait features were identified using a three-stage feature selection framework that includes linear mixed-effects model, ElasticNet-regularized and bootstrap analysis. To explore intra-group variability within the LOPD cohort, we performed a clustering analysis. Based on the selected features and their weighted temporal changes, we developed a Pompe Mobility-Derived Progression Index (Pompe-MDPI) by training a Linear Discriminant Analysis (LDA) to discriminate between control and LOPD data. We calculated the Minimum Clinically Important Difference and compared its performance against the 6MWT distance.

**Results:**

24 LOPD and 39 healthy controls were included in the study. 46 gait features were found to significantly differentiate individuals with LOPD from controls (Holm-corrected *p* < 0.05), comprising 16 from trunk and pelvis joints, 18 from lower limb joints, 4 from force profiles, and 8 from EMG.Hierarchical clustering analysis revealed two distinct subgroups within the LOPD cohort, based on nine gait features. The computed Pompe-MDPI successfully discriminated between LOPD and healthy controls (AUC = 0.95), outperforming the 6MWT distance (AUC = 0.84). The Pompe-MDPI was also strongly associated with the 6MWT (*p* < 0.0001) and demonstrated significant change over time in the LOPD group (*p* = 0.02).

**Conclusions:**

The Pompe Mobility-Derived Progression Index (Pompe-MDPI) was developed and validated as a sensitive biomarker of disease progression. Longitudinal analysis demonstrated that Pompe-MDPI captured gait deterioration over one year, outperforming traditional measures like the six-minute walk test in sensitivity. These findings support the use of wearable gait analysis as a clinically meaningful, scalable tool for monitoring motor function in LOPD, with implications for both patient care and therapeutic trials.

**Supplementary Information:**

The online version contains supplementary material available at 10.1186/s12984-026-01898-8.

## Introduction

Pompe disease is a rare genetic neuromuscular disorder caused by pathogenic variants in the *GAA* gene, resulting in reduced or absent activity of the lysosomal enzyme acid alpha-glucosidase (GAA) [1]. This enzymatic deficiency leads to glycogen accumulation in various tissues, particularly affecting skeletal, cardiac, and smooth muscles, as well as, to a lesser extent, the central nervous system [2]. Clinically, Pompe disease is classified by age of symptom onset into infantile-onset (IOPD) and late-onset Pompe disease (LOPD) [3]. LOPD manifests any time after the age of one year and is characterized by a slowly progressive weakness of limb-girdle, axial, and respiratory muscles and absence of cardiomyopathy [4]. This weakness results in a wide spectrum of functional impairment, ranging from non-ambulant ventilator-dependent individuals to those who maintain independent ambulation and respiratory function [5].

In LOPD, the distribution of weakness typically involves the paraspinal, abdominal, gluteal, and posterior thigh muscles, often sparing distal muscle groups until later stages [4]. This pattern produces a characteristic gait phenotype, frequently described in clinical settings as waddling or myopathic gait often related to hip abductors weakness. Several cross-sectional studies have begun to quantify these gait abnormalities and reported reduced gait velocity, increased pelvic motion, and reduced hip and knee movements in LOPD, using both spatiotemporal and 3D kinematic analysis [6, 7]. Recent studies extended these observations to include compensatory trunk postures and impaired balance control during walking and standing [8–10]. Despite these advances, prior studies have been limited by small cohorts, cross-sectional designs, and reliance on high-cost lab-based technologies. Critically, no study has evaluated whether gait features can sensitively track disease progression over time or serve as clinically meaningful markers of change in longitudinal follow-up.

Enzyme replacement therapy (ERT) remains the standard of care for Pompe disease [3]. In LOPD, current guidelines recommend initiating ERT upon objective detection of skeletal or respiratory involvement, typically confirmed by clinical examination or spirometry [3]. However, in many individuals, weakness remains subclinical until substantial muscle damage has occurred, delaying treatment initiation and diminishing therapeutic benefit. Studies have consistently shown that early initiation of ERT is associated with improved functional and respiratory outcomes [11, 12]. Therefore, international guidelines emphasize the importance of carefully monitoring presymptomatic individuals using functional assessments [3, 13, 14]. These early changes are often too mild to be picked up by current standard tests, such as manual muscle testing, making more sensitive and objective tools increasingly important.

This need is especially pressing in the context of recent and ongoing clinical trials evaluating next-generation therapies including improved ERT formulations and gene therapy which have struggled to demonstrate superiority over existing treatments [15, 16]. One reason may be the limited sensitivity of traditional outcome measures such as the six-minute walk test (6MWT) and forced vital capacity (FVC), which may fail to capture modest but meaningful changes in motor performance [17, 18]. The 6MWT performance has been shown to be significantly influenced by non-neuromuscular factors such as age and cardiopulmonary function, further complicating its interpretation as a disease-specific marker [19, 20].

Historically, gait analysis has been restricted to specialized laboratory settings due to its technical complexity. However, recent technological advances have enabled the use of portable, sensor-based systems that can measure spatiotemporal parameters, joint kinematics, muscle activation, and plantar pressure with high fidelity [21]. These tools offer new opportunities for objective, scalable, and longitudinal monitoring of neuromuscular diseases.

Therefore, the purpose of this study is to develop and validate novel gait-based outcome measures for monitoring disease progression in individuals with LOPD. We hypothesize that specific gait parameters are sensitive indicators of disease progression in LOPD and that these features can be integrated using artificial intelligence into a disease-specific Mobility-Derived Progression Index (Pompe–MDPI), which will demonstrate greater sensitivity than the 6-minute walk distance (6MWD) in detecting longitudinal changes. Additionally, we expect to identify distinct gait phenotypes within the LOPD group, reflecting variability in disease severity.

## Methods

### Study design

A longitudinal, prospective study involving individuals with genetically confirmed LOPD, designed to evaluate whether gait analysis using an easy-to-wear portable device can serve as a reliable outcome measure to distinguish patients from healthy controls and to detect meaningful changes over time.

Inclusion criteria were: (1) a confirmed genetic diagnosis of LOPD with symptom onset after the age of one year; (2) the ability to perform 6MWT with or without the use of assistive walking devices such as a cane or walking stick; and (3) willingness to participate in all study assessments. Non-ambulant individuals were excluded.

The study enrolled both individuals with LOPD and age- and gender-matched healthy controls. Ethical approval was obtained from the Ethics Committee of the Hospital Nacional de Parapléjicos in Toledo (Spain) and the University of Florida (USA). Written informed consent was obtained from all participants.

Most assessments were conducted during the annual meetings of the Spanish Association of Patients with Pompe Disease, although some participants were evaluated at their respective reference hospitals. The sample size was determined based on the availability of eligible individuals and their willingness to participate. All participants were assessed using the Ephion Mobility system (www.ephion.health) and completed various functional assessments, including a free walking test (covering a distance of 10–15 m at a self-selected pace) and the 6MWT. Healthy participants also performed test walking at self-selected slow, comfortable and fast speed. To ensure the findings reflected disease-specific gait abnormalities and were not confounded by age- or walking speed-related factors, LODP and control groups were matched for both age and gait velocity using a 1:1 individual-level matching greedy nearest-neighbor algorithm.

### Gait analysis technology

Gait assessments were conducted using the Ephion Mobility system, a multi-sensor wearable platform. The setup included five inertial measurement units (Movesense, Vantaa, Finland) placed on the shanks, thighs, pelvis, and chest, along with insoles equipped with both inertial and plantar pressure sensors (Moticon, Munich, Germany). In addition, surface electromyography (EMG) sensors (Myontec, Kuopio, Finland) were placed on the thighs to capture muscle activation. All sensors were synchronized using the Ephion Mobility software, which operated via a smartphone interface. This configuration enabled the acquisition of a comprehensive set of gait features, including: (1) *Kinematic data*: angular motion of the foot, ankle, knee, hip, pelvis, and trunk; (2) *Spatiotemporal metrics*: gait velocity, double support time, stance time, stride length, and cadence; (3) *Plantar pressure variables*: vertical ground reaction force and center of pressure (COP) trajectory and; (4) *Muscle activation*: EMG signals from the quadriceps, hamstrings, and gluteal muscles.

Participants wore the full system while performing the walking tasks described previously. This same system has been successfully used in prior studies to characterize gait impairments in other neuromuscular conditions, including Duchenne muscular dystrophy (DMD), Becker muscular dystrophy (BMD), Myotonic Dystrophy (MD) and various types of peripheral neuropathies such as chronic inflammatory demyelinating polyneuropathy (CIDP) [22, 23].

### Data analysis

The data collected across the entire walking tests was segmented into gait cycles. A gait cycle represents a step, beginning and ending when the same foot contacts the ground. The analysed data corresponds to the mean of all gait cycles of the test. Each gait cycle was time-normalized to 100 points, representing 0–100% of the cycle, and then averaged to obtain the mean gait pattern. This normalization allowed direct comparison of gait characteristics across tests performed at different walking speeds. More information on data recording and processing can be found in previously published studies using this technology [22].

To analyse the mean gait cycles, several relevant features were identified including morphological features, focused on the shape and timing on the signals (such as peak and valley values, and durations of key phases within the cycle) and statistical features, which described the variability and distribution of the signals (Supplementary Table 1). Moreover, we calculated the Gait Profile Score (GPS), a comprehensive measure of gait deviations using kinematic and matched control data as the normative reference [24].

### Statistical analysis

Descriptive statistics were calculated for demographic and baseline characteristics. Independent t-tests were used for continuous variables and chi-squared tests for categorical variables. To identify differences in gait features between the control and LOPD groups, we employed a three-stage feature selection framework. First, a linear mixed-effects (LME) model estimated the impact of gait features on LOPD pathology while accounting for subject-level variability through random intercepts. Second, ElasticNet-regularized regression was applied to the LME model residuals, using a weighted design matrix for robust feature selection that addresses multicollinearity. Third, a 100-iteration bootstrap analysis identified stable features, retaining those selected in > 80% of iterations. This pipeline addressed temporal dependencies, regularization bias, and model instability, maintaining Type I error control. The resulting set of 50 features was then subjected to Holm correction for multiple comparisons, with significance set at a corrected *p* < 0.05.

To explore intra-group variability within the LOPD cohort, we performed a multi-step clustering analysis. First, each sample’s missing values were imputed using the mean value from the five nearest neighbours found in the dataset and Principal Component Analysis (PCA) was applied to extract major variance components and to compute contribution scores for each feature. All feature values were then standardized using z-scores based on the control group distribution. Hierarchical clustering was performed using complete linkage and cosine distance as the similarity metric. To define the optimal feature set for clustering, we selected features exceeding a contribution threshold that maximized the silhouette score while ensuring representation from at least five distinct sensor domains, thus capturing a holistic view of gait patterns. Cluster quality was assessed based on clinical interpretability, and Kruskal–Wallis tests with post-hoc comparisons were used to evaluate differences in key gait features between clusters. Demographic differences between clusters were also described.

Based on the selected features and their weighted temporal changes, we developed a Pompe Mobility-Derived Progression Index (Pompe-MDPI). The LOPD sample was randomly divided into a training set (70% of participants) to develop the model and a test set (30%) to then validate it on unseen data. A Linear Discriminant Analysis (LDA) was fitted to the training data, and the Pompe-MDPI was defined as each participant’s score on the resulting single discriminant function. Model performance was evaluated on the test set using Receiver Operating Characteristic (ROC) analysis. To enhance interpretability of the model’s predictions, SHAP (SHapley Additive exPlanations) analysis was used to quantify the contribution of each feature to the classification outcome [25].

The Minimum Clinically Important Difference (MCID) was estimated using a distribution-based approach, defined as 0.5 times the standard deviation of baseline values—representing a moderate effect size. As a reference, we used the 6MWT as gold standard, where a change of ≥ 29 m was considered clinically meaningful [26]. Logarithmic changes were used to compare individual trajectories, and LME was applied to both LOPD and control groups to evaluate the longitudinal evolution of the biomarker. All analysis were performed using a custom Python (v3.11) pipeline developed by Ephion.

### Data availability

Raw data of the study is available for further research from the corresponding author on reasonable request.

## Results

### Study cohort

A total of 24 individuals with LOPD were included in the study and completed a baseline assessment. Of these, 14 participants underwent a one-year follow-up, and five were also assessed at two years. Additionally, 39 age- and gait velocity–matched healthy individuals were included as controls. Among the control group, 13 subjects completed a same-day retest, and 8 underwent a follow-up assessment within one year. In total, the dataset comprised 43 walking tests individuals with LOPD and 60 from healthy controls. Two patients completed the test using an assistive device, resulting in a total of five trials performed with a walking aid. Baseline demographic and clinical characteristics of all participants are summarized in Table 1.

**Table 1:** Demographic and Spatiotemporal characteristics of the study sample with LOPD patients and age and velocity matched healthy controls

		Grouped by Pathology			
		Missing	CTL	Pombe	P-value
n			60	43	
AGE, mean (SD)		0	52.5 (14.4)	54.4 (16.1)	0.542
SEX, n (%)	Female	0	36 (60.0)	20 (46.5)	0.248
	Male		24 (40.0)	23 (53.5)	
ERT, n (%)	No	60		2 (4.7)	1.000
	Yes			41 (95.3)	
VELOCITY, mean (SD)		0	1.3 (0.2)	1.2 (0.3)	0.083
CADENCE, mean (SD)		0	59.4 (7.3)	53.4 (5.2)	<0.001
STRIDE LENGTH NORM, mean (SD)		0	1.3 (0.2)	1.4 (0.3)	0.536
DOUBLE SUPPORT, mean (SD)		2	25.5 (6.0)	27.7 (5.2)	0.048
STANCE TIME, mean (SD)		1	62.7 (2.2)	64.0 (2.5)	0.006
6MWD, mean (SD)		4	3.6 (1.0)	4.8 (1.0)	<0.001
GPS Right, mean (SD)		3	632.6 (95.3)	437.5 (107.7)	<0.001
RETEST, n (%)	0	0	39 (65.0)	24 (55.8)	0.416
	1		14 (23.3)	14 (32.6)	
	2		5 (8.3)	5 (11.6)	
	3		2 (3.3)		

Values are presented as mean (standard deviation) for continuous variables and n (%) for categorical variables. Independent t-tests were used for continuous variables and chi-squared tests for categorical variables

Abbreviations: ERT, enzyme replacement therapy; GPS, Gait Profile Score; 6MWD, 6-minute walk distance

### LOPD vs. control gait characteristics

LOPD participants covered significantly less distance than healthy controls in the 6MWT. When comparing spatiotemporal gait parameters between LOPD and healthy participants matched for age and gait velocity, LOPD participants exhibited significantly lower cadence and spent more time in the stance phase, leading to an increased duration of double support. Moreover, the GPS was significantly higher in the LOPD group, indicating greater deviation from the normative kinematic reference. No significant differences were observed in gait velocity or stride length, as detailed in Table 1.

**Fig. 1:**
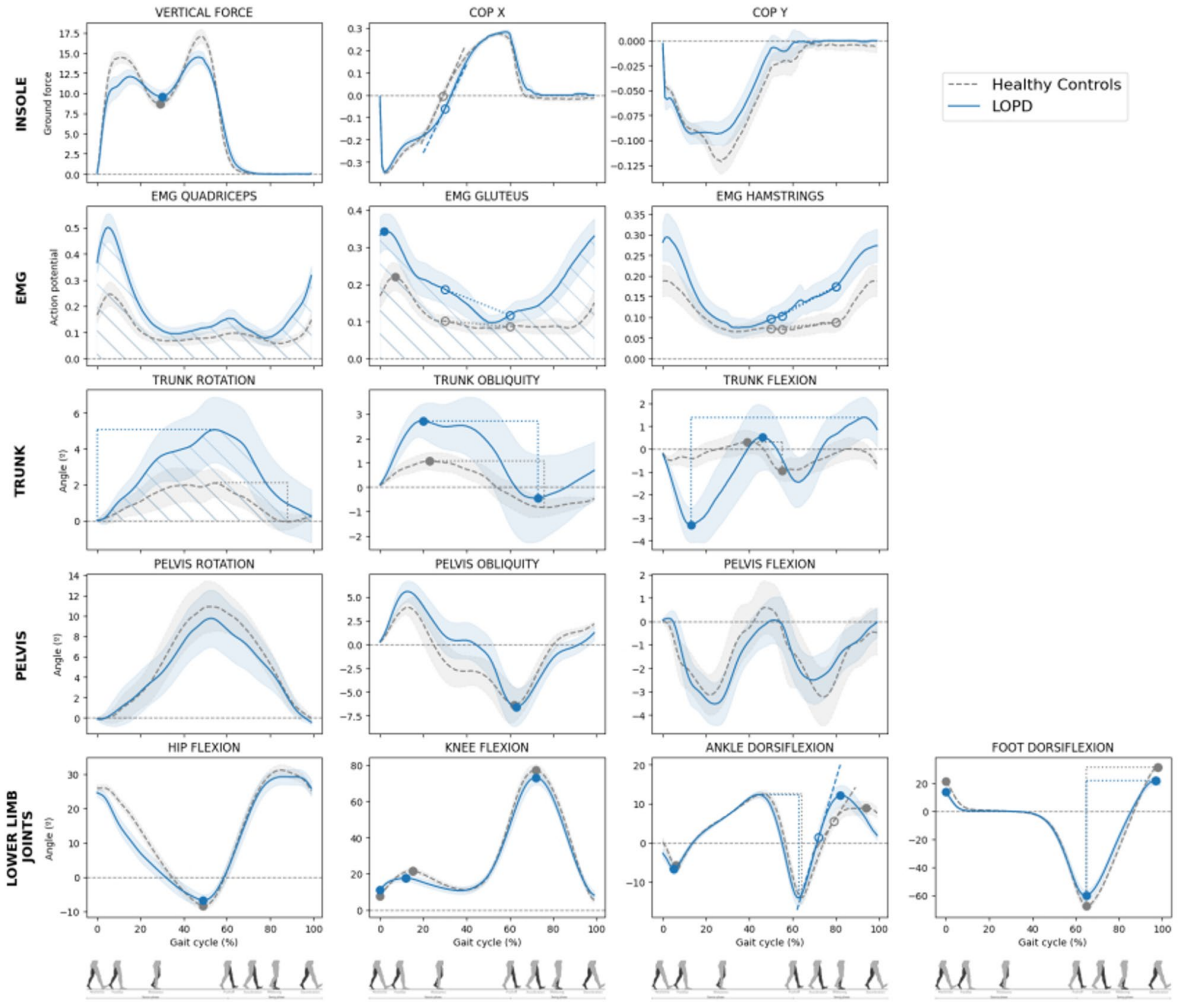
Mean gait cycle curves across multiple biomechanical domains: kinematics, EMG and ground reaction forces, in LOPD participants and healthy controls. Representative average waveforms (mean ± 95% confidence interval) for each group are shown across the gait cycle (0–100%). Blue solid lines indicate data from participants with late-onset Pompe disease (LOPD), and dashed gray lines represent healthy controls. Shaded regions denote 95% confidence intervals for each group. Selected significant features identified by the three-stage pipeline are highlighted within the profiles for illustrative purposes. Not all significant features could be displayed due to overlapping representations or the absence of a clear waveform expression. Gait phases are illustrated at the bottom of the figure for reference

Mean curve profiles for both LOPD participants and healthy controls are presented in Fig. 1. From the 50 selected gait features by the three-stage feature selection framework, LME identified 46 features that significantly differed between groups: 16 from trunk and pelvis sensors, 18 from lower limb joint sensors, 4 from plantar pressure sensors, and 8 from surface EMG. LOPD participants exhibited increased motion in all three planes of trunk movement—flexion-extension, internal-external rotation, and lateral tilt. Trunk-related features, including range of motion (ROM), peak and valley values, prominence, and movement speed variability, were significantly elevated in the LOPD group compared to controls.

Pelvic flexion-extension and internal rotation were similar between groups, but LOPD participants showed altered lateral tilt with compensatory contralateral pelvic drop during stance, which resulted in a significantly reduced valley width during push-off.

At the hip, LOPD participants experienced a prolonged period of reduced hip flexion during the initial phase of the stance, as evidenced by a significantly wider valley width.

Knee kinematics revealed reduced flexion during the stance phase in LOPD participants, beginning with a significantly more flexed knee at initial contact, and reaching peak flexion significantly earlier. In some cases, knee flexion was completely absent during this stage. During the swing phase, LOPD participants presented a significantly narrower flexion duration.

LOPD participants showed a significantly greater ROM throughout the gait cycle, characterized by increased dorsiflexion and a steeper dorsiflexion slope during the swing phase. Peak dorsiflexion occurred significantly earlier, leading to a more plantarflexed position at ground contact.

In contrast, foot flexion ROM was significantly reduced in LOPD patients, with both plantarflexion and dorsiflexion values diminished. Consequently, foot positioning at initial contact was also affected, with LOPD participants exhibiting reduced foot dorsiflexion at heel strike. The described kinematic alterations also impacted ground reaction forces (GRF). LOPD patients present a visibly flatter force curve, characterized by lower peak forces at heel contact and toe-off, and increased force values during mid stance (20–40% of the gait cycle), corresponding to the single-limb support phase as the body transitions from absorption to propulsion. Feature analysis revealed a significant reduction in the entropy of the GRF, which measures the complexity of the force distribution pattern, accompanied with reduced prominence and width on the first valley. Foot COP progression also differed: LOPD participants presented a slower transition from heel to toe, highlighted by a significantly shallower slope value. EMG analysis revealed increased and prolonged muscle activation in gluteus, hamstrings, and quadriceps throughout the gait cycle in LOPD participants, with earlier, higher, and prolonged activation observed at initial contact and swing phase, corresponding to the onset of hip and knee flexion for limb advancement. These differences were supported by statistical analysis of EMG-derived features. Significant group differences were found in the area under the curve, signal standard deviation, prominence on the first peak of the gluteus, mean stance-phase values for the hamstrings, and peak amplitudes between 40 and 80% of the cycle for both the hamstrings and gluteus muscles.

### LOPD clustering

**Fig. 2:**
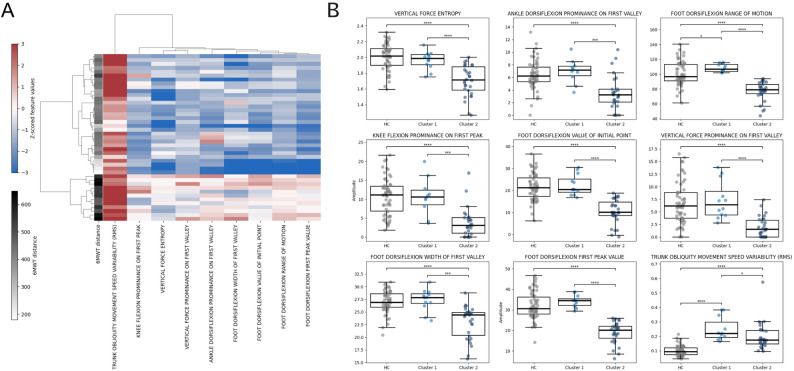
Cluster-based stratification of LOPD participants based on key gait features. **A** Heatmap showing hierarchical clustering of LOPD participants (rows) and selected gait features (columns) using z-scored values. Additionally, a grayscale is added showing the distance walked in the 6MWT. Warmer colors represent higher z-scored values; cooler colors represent lower values. **B** Boxplots displaying group differences across healthy controls (HC), Cluster 1, and Cluster 2 for nine selected gait features. Statistical comparisons were performed using Kruskal–Wallis tests with post-hoc comparisons. Significance levels are indicated as follows: *p* < 0.05 (*),p < 0.01 (**), p < 0.001 (***), and *p* < 0.0001 (****)

Hierarchical clustering analysis revealed two distinct subgroups within the LOPD cohort, based on nine gait features selected, which maximized the clustering silhouette score (Fig. 2B). Of these features, four were derived from foot kinematics, two from insole pressure data, one from knee joint motion, one from the ankle, and one from trunk dynamics. A heatmap of the selected features highlights distinct patterns in the key gait features across clusters, visually supporting the separation of the two subgroups (Fig. 2A). Although clustering analysis highlighted these nine features, differences between the clusters were observed in other gait parameters, such as hip and knee flexion angles as shown in Supplementary Fig. 1, which shows the mean cycle profiles for both LOPD clusters and healthy controls. Table 2 summarizes the demographic and spatiotemporal characteristics of each subgroup.

**Table 2:** Demographic and Spatiotemporal characteristics of the study sample by cluster

		Grouped by Cluster			
		Missing	Cluster 1	Cluster 2	P-value
n			12	31	
AGE, mean (SD)		0	40.5 (17.1)	59.8 (12.2)	0.003
SEX, n (%)	Female	0	4 (33.3)	16 (51.6)	0.461
	Male		8 (66.7)	15 (48.4)	
ERT, n (%)	Yes	0	12 (100.0)	29 (93.5)	1.000
	No			2 (6.5)	
VELOCITY, mean (SD)		0	1.6 (0.2)	1.1 (0.2)	<0.001
CADENCE, mean (SD)		0	58.3 (6.6)	51.5 (2.9)	0.004
STRIDE LENGTH NORM, mean (SD)		0	0.9 (0.1)	0.7 (0.1)	<0.001
DOUBLE SUPPORT, mean (SD)		1	23.8 (4.3)	29.3 (4.7)	0.002
STANCE TIME, mean (SD)		0	62.2 (2.0)	64.7 (2.3)	0.002
6MWD, mean (SD)		0	561.2 (59.9)	389.6 (80.2)	<0.001
GPS Right, mean (SD)		4	4.5 (0.4)	5.0 (1.2)	0.053

Values are presented as mean (standard deviation) for continuous variables and n (%) for categorical variables. Independent t-tests were used for continuous variables and chi-squared tests for categorical variables

Abbreviations: ERT, enzyme replacement therapy; GPS, Gait Profile Score; 6MWD, 6-minute walk distance

Cluster 1 consisted of younger participants whose spatiotemporal parameters more closely resembled those of healthy controls. Compared to cluster 2, LOPD participants in cluster 1 covered a significantly longer distance in the 6MWT, with higher gait velocity, cadence, and stride length. They also demonstrated significantly lower double support and stance times, indicating a more stable and efficient gait pattern. In contrast, cluster 2 revealed a global reduction in dynamic range and adaptability during gait, characterized by slower movement patterns and broader gait cycle peaks. As shown in the boxplots in Fig. 2B, participants in cluster 1 exhibited greater trunk obliquity motion, indicated by a significantly higher movement speed variability, compared to cluster 2 and HC. During the landing phase, cluster 2 displayed compensatory mechanisms characterized by contacting the ground with a less flexed foot, resulting in lower ground impact compared to Cluster 1 and HC. This is reflected in significantly reduced prominence on the ankle, reduced foot flexion value at initial point and first-peak values, and reduced entropy on the vertical force. In the stance phase, both knee flexion and vertical force profiles remained constant, associated with reduced knee flexion prominence and a less prominent valley in the vertical force signal. During the swing phase, participants in cluster 2 showed reduced flexion at foot flexion, evidenced by a lower ROM of the foot segment, contrary to what was observed in cluster 1, where participants had significantly higher foot flexion ROM.

### Pompe-MDPI: the Pompe mobility-derived progression index

**Fig. 3:**
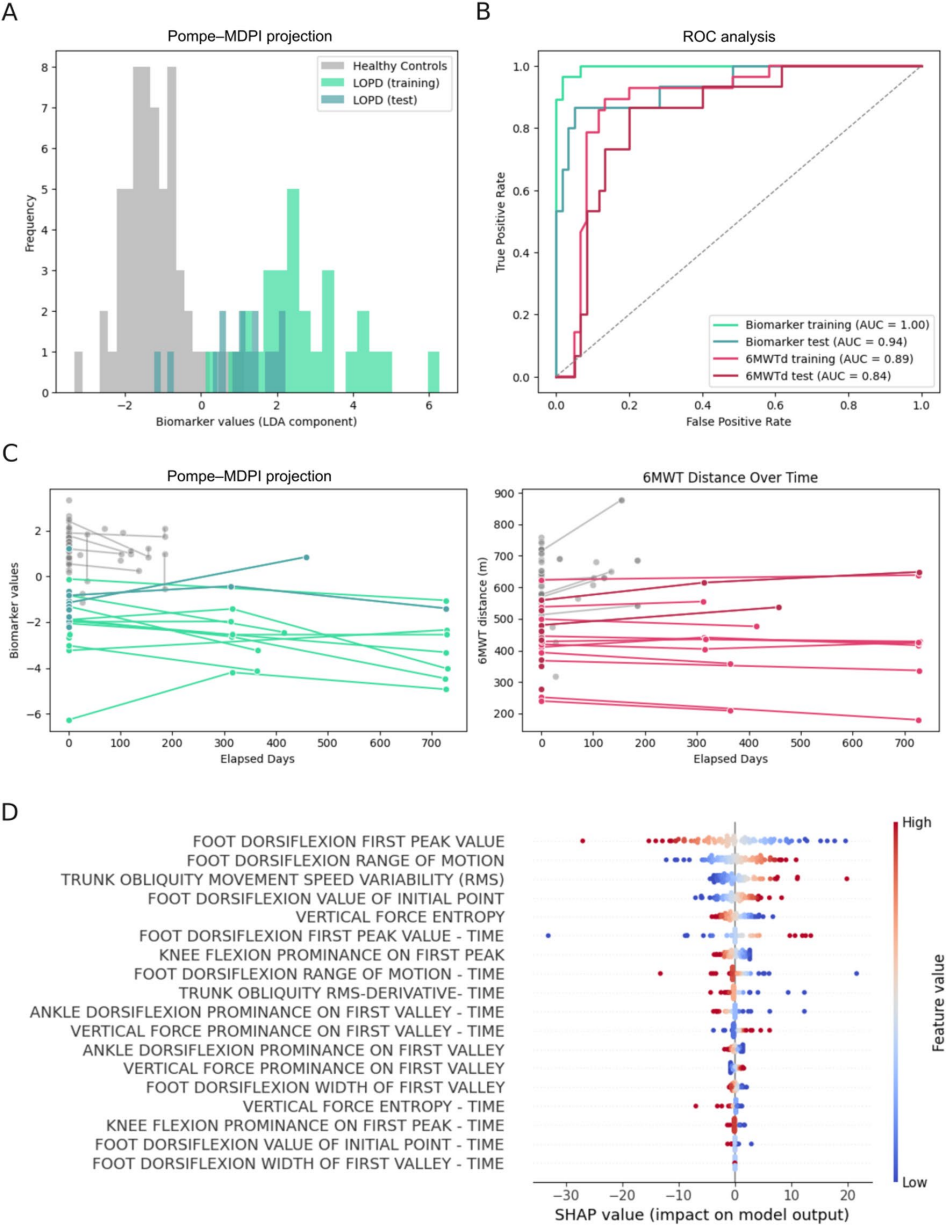
Development, performance, and interpretation of the Mobility-Derived Progression Index (Pompe – MDPI). **A** Histogram showing the distribution of the Pompe Mobility-Derived Progression Index (Pompe – MDPI) across healthy controls (grey), and LOPD participants split into training (greenish blue) and test (green) sets. The x-axis shows the values of the LDA component (the monitoring biomarker) whereas the y-axis indicates the frequency (number of samples) for each bin. **B** ROC curves comparing the classification performance of the Pompe – MDPI against the 6-minute walk test (6MWT) in distinguishing LOPD from healthy controls. **C** Longitudinal evolution of both the Pompe – MDPI (left) and 6MWT distance (right) over time for individual healthy controls (grey), and LOPD participants split into training (greenish blue) and test (green) sets. **D** SHAP summary plot showing the most influential features in the Pompe – MDPI. Features are ordered by importance; each dot represents a sample, with color indicating feature value (blue: low, red: high), and x-axis indicates SHAP value (impact on model output). Positive SHAP values contribute to classifying a sample as LOPD; negative values contribute toward healthy control classification

Pompe-MDPI: successfully discriminated between LOPD participants and healthy controls. Figure 3A displays the distribution of the biomarker values (LDA component) for the two subgroups. The histogram demonstrates a clear separation with minimal overlap: healthy control samples were clustered around positive values, whereas LOPD training samples were mainly distributed in the negative values. The overlap between training and validation LOPD samples supports the model’s ability to generalize to unseen data. Figure 3B shows the ROC analysis for both training and test subsamples of LOPD. The model achieved excellent classification performance, with an area under the curve (AUC) of 1.0 for the training set and 0.95 for the test set indicating that the computed biomarker successfully discriminates between these two populations using the selected features. These values are higher than those obtained with the 6MWT distance (training AUC 0.89 and test AUC 0.84). Figure 3C displays the SHAP analysis, highlighting the features contributing most strongly to the LDA model. This analysis provides insights into how specific features contribute to the Pompe-MDPI. Key contributors included the first peak value of foot dorsiflexion, dorsiflexion range of motion and trunk obliquity movement speed variability (RMS). High values of the foot dorsiflexion first peak (shown in red) were associated with higher SHAP values, indicating a strong contribution to classifying control subjects. Conversely, lower values (in blue) were associated with classification as LOPD. The biomarker showed a moderate but statistically significant correlation with the distance covered in the 6MWT (*r* = 0.47, *p* < 0.0001), supporting its clinical relevance.

### Longitudinal follow-up of participants

**Fig. 4:**
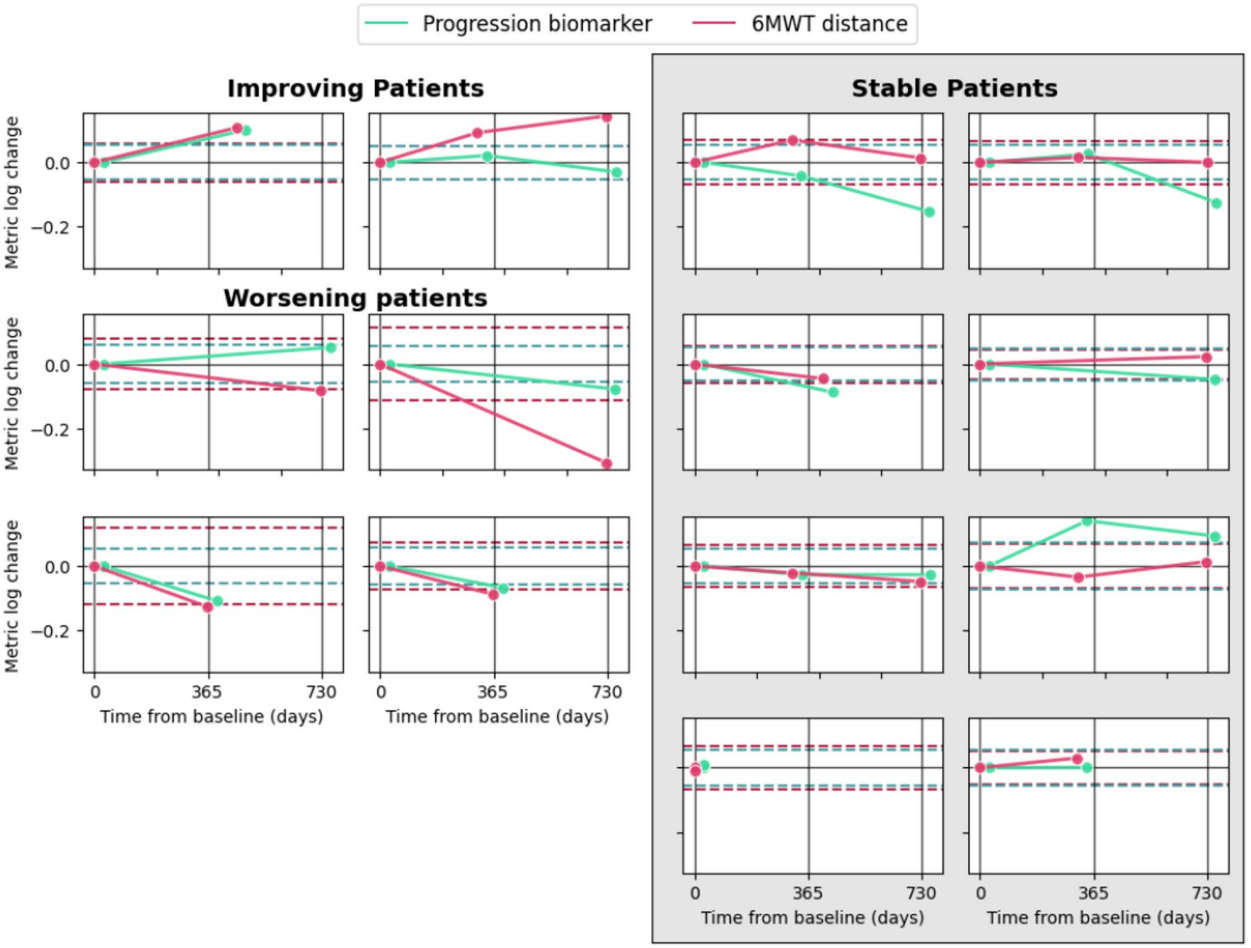
Individual longitudinal changes in the Pompe - MDPI and the 6-minute walk test (6MWT) distance across patient categories over a 2-year period. Line graphs depict the log change from baseline in Pompe–MDPI (green) and 6MWT distance (red). Patients are classified as improving (top-left panels), stable (right panels), or worsening (bottom-left panels) according to the 6MWT distance. The y-axis represents metric log change from baseline, while the x-axis shows time in days. Horizontal dashed lines indicate clinically significant thresholds: green lines represent the minimal clinically important difference (MCID) for the Pompe – MDPI, and red lines represent the log-transformed value of 29 m for 6MWT, considered a meaningful functional change

Using the distribution-based approach detailed in methods, we estimated an MCID of 0.97 for the Pompe-MDPI, which resulted in a 70.0% agreement with 6MWT distance when assessing progression. Of the four subjects labelled as progressors by the 6MWT distance, three were also classified as progressors by Pompe-MDPI. Among the eight participants classified as stable by the 6MWT, four were identified as progressors by the Pompe-MDPI. This discrepancy may reflect the biomarker’s higher sensitivity to subtle gait changes that precede measurable declines in 6MWT performance, suggesting its potential for earlier detection of disease progression. Figure 4 illustrates individual trajectories for both the 6MWT distance and the computed biomarker. Fourteen LOPD participants had at least one follow-up assessment, and longitudinal data from healthy controls were also included. When analysing all longitudinal data (Fig. 4), distinct patterns emerged between groups: healthy controls showed no significant change over time (*p* = 0.10), while LOPD participants demonstrated a significant decrease in Pompe-MDPI, indicating worsening on the gait patterns (*p* = 0.02).

## Discussion

In this study, we evaluated gait in individuals with LOPD and age- and velocity-matched healthy controls using a multi-sensor, portable system. Our findings enabled the identification of a disease-specific gait signature that not only distinguishes LOPD from healthy individuals but also reflects a spectrum of disease severity. Importantly, we showed that key gait parameters evolve over time and can be captured longitudinally, supporting the development of a disease-specific Mobility-Derived Progression Index (Pompe-MDPI) that may serve as a sensitive functional biomarker in both clinical practice and clinical trials.

We have used an easy to wear portable device consisting of a combination of sensors, thighs and insoles that collected multiple parameters of gait, including spatio-temporal features, but also kinematics, muscle activation and plantar pressure. Through this multimodal approach, we characterized a consistent set of abnormalities, that we could consider as the “Pompe gait pattern fingerprint” that were common to most of the participants. This included reduced distance in the 6MWT, shorter stride length and lower cadence, increased double support time, exaggerated trunk motion in all planes, increased lateral pelvic tilt, early and elevated activation of posterior thigh and gluteal muscles, reduced knee flexion during stance, and exaggerated ankle dorsiflexion during swing compared to controls. These alterations resulted in an abnormal force distribution across the gait cycle. Similar patterns have been partially described in earlier gait studies using 2D and 3D systems, as well as force plate data, though ours is the first to integrate all modalities in a portable and longitudinal framework [6, 7, 9].

The same system has been recently used to study other diseases, such as DMD, BMD, MD and CIDP. Compared with DMD and BMD, which also present with proximal muscle weakness, LOPD shares some overlapping gait characteristics, most notably, reduced or absent knee flexion during the stance phase and increased compensatory trunk movement [27, 28]. However, several features clearly distinguish LOPD from these conditions. In contrast to DMD, which typically presents with increased anterior pelvic tilt, reduced hip extension, and exaggerated hip and knee flexion during swing, LOPD participants maintain relatively preserved hip swing mechanics but show more pronounced alterations at the ankle [22]. Specifically, LOPD is characterized by increased and earlier ankle dorsiflexion during swing, reduced foot flexion at initial contact and throughout swing, and a flatter, less complex vertical force profile, reflecting altered ground contact and reduced propulsion. Compared to BMD, which shows reduced hip extension, plantarflexed ground contact, and greater pelvic motion at toe-off, LOPD differs in both foot strike dynamics and vertical force modulation. Moreover, while increased EMG activity is present in all three diseases, LOPD exhibits distinct temporal activation patterns of the gluteus, hamstrings, and quadriceps across the gait cycle. Together, these findings support the existence of disease-specific gait “fingerprints,” measurable using wearable sensor technology, which may aid in differential diagnosis and more tailored monitoring strategies in clinical practice.

Although the overall gait pattern in LOPD was relatively consistent across participants, we observed a degree of variability that could be related to the heterogeneous disease stages represented in our cohort, from early to more advanced progression. This variability provided a unique opportunity to explore which abnormalities in the gait pattern may emerge earliest, with potential relevance for identifying subclinical muscle involvement in genetically confirmed but functionally presymptomatic individuals. We observed early abnormalities in the trunk motion and knee flexion mechanics, suggesting an early imbalance in the strength and coordination between the psoas, gluteal, and posterior thigh muscles versus the quadriceps, aligning with previous reports of early selective muscle involvement in LOPD disturbance in the strength balance between psoas-gluteus and posterior muscles of the thigh and quadriceps [29]. Such impairments often manifest clinically as reduced capacity to rise from the floor or a chair, or to climb up stairs. These findings are also consistent with imaging studies demonstrating early fat replacement in the paraspinal, gluteal, and proximal thigh muscles supporting the physiological basis of these gait alterations [30, 31]. As disease progresses, additional gait parameters become affected in a more complex and multidimensional fashion, as described in our analysis, defining a set of parameters that can be followed up in clinics to detect disease progression.

Previous studies have assessed gait in LOPD using a variety of tools, ranging from static posture assessments to comprehensive 3D gait laboratory setups. Our findings are consistent with these reports, particularly regarding reduced spatiotemporal performance. Several groups have documented significantly decreased walking distance, cadence, and gait velocity in LOPD participants as observed in our cohort [8, 10]. A consistently reported feature across studies is the prolonged stance phase, which likely reflects a compensatory adaptation aimed at improving stability in due to muscle weakness or balance challenges [7, 20]. Trunk kinematics during gait appear to change in a non-linear manner across disease progression in late-onset Pompe disease. In earlier and intermediate stages, weakness of axial and pelvic stabilizing muscles, including paraspinal, abdominal, and gluteus medius and minimus muscles, may lead to increased trunk movements as a compensatory strategy to preserve balance and forward progression in the presence of pelvic instability. This pattern is consistent with previous reports describing exaggerated trunk excursions in patients with greater functional impairment. In contrast, in more advanced stages, as suggested by Cluster 2, further involvement of proximal muscles such as the gluteus maximus, psoas, thigh musculature, and upper-limb proximal muscles may limit the ability to actively modulate trunk motion. Consequently, trunk inclination may appear reduced, reflecting a more rigid gait pattern rather than effective compensation. These findings suggest a shift from compensatory trunk hypermobility to reduced trunk mobility as axial and proximal muscle weakness progresses, highlighting the importance of considering disease stage when interpreting trunk kinematics in LOPD. Early reduction in knee flexion during stance and alterations in ankle flexion-extension cycles have also been reported in the past, associated to reduced strength of gluteus, psoas and posterior thigh muscles, leading to an impaired activation of these muscles during gait [7, 8].

The longitudinal design of our study, combined with the application of artificial intelligence techniques, enabled the identification of key gait parameters that changed over a one- to two-year period in several LOPD participants. Based on these dynamic features, we developed the Pompe Mobility-Derived Progression Index (Pompe-MDPI) that simplifies gait analysis and facilitates standardized comparisons across participants. Notably, many individuals in our cohort exhibited a decline in this score over time. Although the Pompe-MDPI still needs validation in broader studies, our findings suggest it holds promise as a sensitive tool to detect subtle progression in LOPD in participants treated with ERT, where disease progression is typically slow and difficult to detect by conventional outcome measures [32]. Traditional tests such as the 6MWT may take years to reflect meaningful change in this population, complicating the design and interpretation of clinical trials [18, 23]. In contrast, the Pompe-MDPI demonstrated the ability to capture short-term changes in motor function, indicating its potential as a more responsive and clinically useful outcome measure for both research and real-world monitoring. Given these advantages, the Pompe-MDPI may complement or even outperform current functional tests in future therapeutic trials.

The main limitation of our study is the relatively small sample size. LOPD is a rare condition with an estimate number of 160 participants living in Spain. Our cohort of 24 participants represents approximately 15% of them, which, while modest in absolute terms, reflects strong representation within the context of a rare disease [12]. The inclusion of participants performing the test with a walking aid may influence gait kinematics. However, their inclusion reflects real-world clinical gait patterns. The data collected here needs to be validated in a large scale multicentric study before both the gait analysis in general and the Pompe-MDPI in particular, can be accepted for it use as an outcome measure in clinical trials. Although this has not yet been tested, the approach is based on standardized sensor data and reproducible algorithms, which suggests that it could be applicable across different clinical settings and conditions. To minimize potential confounding effects, we employed gait velocity–matched controls, recognizing that walking speed can influence spatiotemporal metrics, joint kinematics, EMG activity, and ground reaction forces. This matching approach helps ensure that the differences observed in our study are more directly attributable to disease-related motor impairments rather than to differences in walking speed alone [33–36].

In summary, our results suggest that wearable gait analysis offers clinically meaningful information about motor performance in LOPD and is sensitive enough to detect changes over relatively short time intervals. This has important implications for both the follow-up of participants in routine care and for evaluating therapeutic interventions in clinical trials. Moreover, the use of a portable platform such as the Ephion Mobility system significantly reduces assessment time and logistical burden, making it feasible to implement gait monitoring not only in the clinic but also remotely, including at participants’ homes prior to hospital visits.

## Supplementary Information

Below is the link to the electronic supplementary material.

Supplementary Material 1

Supplementary Material 2

## Data Availability

Raw data of the study is available for further research from the corresponding author on reasonable request.
