# Supplementary figures and images for: Gait analysis reveals new outcome measures for monitoring disease progression in individuals with late-onset Pompe disease
Source: J Neuroeng Rehabil. 2026 Mar 9;23:149. doi: 10.1186/s12984-026-01898-8 (PMC13147610; doi:10.1186/s12984-026-01898-8)

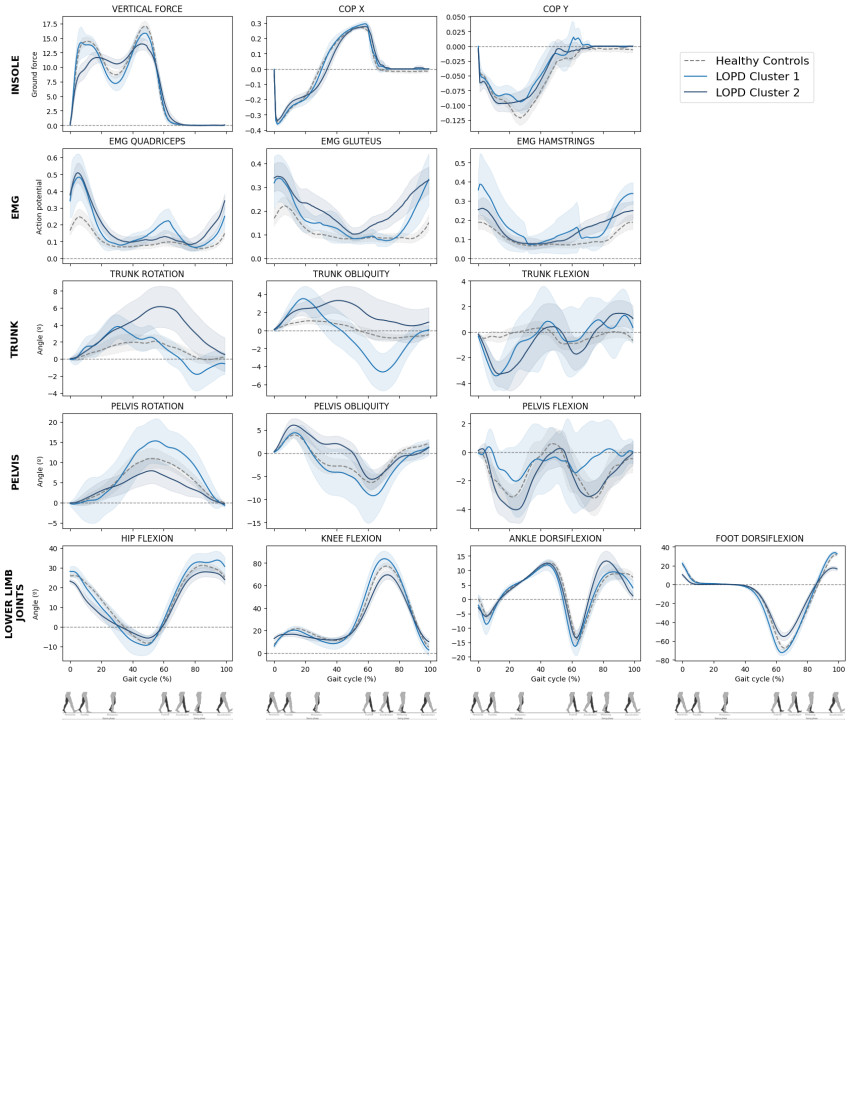

Supplement: Supplementary file 1 — Supplementary Material 1 [file 12984_2026_1898_MOESM1_ESM.jpeg]
