# Supplementary material for: Gait analysis reveals new outcome measures for monitoring disease progression in individuals with late-onset Pompe disease
Source: J Neuroeng Rehabil. 2026 Mar 9;23:149. doi: 10.1186/s12984-026-01898-8 (PMC13147610; doi:10.1186/s12984-026-01898-8)
Supplement: Supplementary file 2 — Supplementary Material 2 [file 12984_2026_1898_MOESM2_ESM.docx]

**Supplementary Table 1.** Detailed description of extracted gait features across biomechanical domains.
It includes all features derived from kinematic, plantar pressure, EMG, and spatiotemporal data. Features are organized by sensor type and corresponding biomechanical profile. For each, the specific feature name and a concise definition are provided.

| **Sensors** | **Variables** | **Features** | **Definition** |
| --- | --- | --- | --- |
| Inertial sensor (movesense) | Hip angle flexion | First peak (amplitude, time, prominence and width) | Maximum point of flexion-extension at stance phase. Value and % of gait cycle when it occurs. |
|  |  | Second peak (amplitude, time, prominence and width) | Maximum point of flexion-extension at swing phase. Value and % of gait cycle when it occurs. |
|  |  | First valley (amplitude, time, prominence and width) | Minimum point of flexion-extension between middle-stance and middle-swing phase. Vale and % of gait cycle when it occurs. |
|  |  | First slope | Slope of signal trajectory between first peak and first valley time-points. |
|  |  | Second slope | Slope of signal trajectory between first valley and second peak time-points. |
|  | Knee angle flexion | First peak (amplitude, time, prominence and width) | Maximum point of flexion-extension between heel-strike and heel-off phase. Value and % of cycle when it occurs. |
|  |  | Second peak (amplitude, time, prominence and width) | Maximum point of flexion-extension during swing phase. Value and % of gait cycle when it occurs. |
|  |  | First valley (amplitude, time, prominence and width) | Minimum point flexion-extension between middle-stance and toe-off phase. Value and % of gait cycle when it occurs. |
|  |  | First slope | Slope of signal trajectory between first valley and second peak time-points. |
|  | Ankle angle flexion | First peak (amplitude, time, prominence and width) | Maximum point of flexion-extension between middle-stance and toe-off phase. Value and % of gait cycle when it occurs. |
|  |  | Second peak (amplitude, time, prominence and width) | Maximum point of flexion-extension during swing phase. Value and % of gait cycle when it occurs. |
|  |  | First valley (amplitude, time, prominence and width) | Minimum point of flexion-extension during heel-strike. Value and % of gait cycle when it occurs. |
|  |  | Second valley (amplitude, time, prominence and width) | Minimum point of flexion-extension between pre-swing and middle-swing phase. Value and % of gait cycle when it occurs. |
|  |  | Range of motion - dorsiflexion | Amplitude of signal between midswing and the end of heel strike. |
|  |  | First slope | Slope of signal trajectory between first valley and first peak time-points. |
|  |  | Second slope | Slope of signal trajectory between first peak and second valley time-points. |
|  |  | Third slope | Slope of signal trajectory between second valley and second peak time-points. |
|  | Foot angle flexion | First peak (amplitude, time, prominence and width) | Maximum point of flexion-extension at swing phase. Value and % of cycle when it occurs. |
|  |  | First valley (amplitude, time, prominence and width) | Minimum point of flexion-extension at swing phase. Value and % of cycle when it occurs. |
|  |  | First slope | Slope of signal trajectory between first value and first peak time-points. |
|  |  | slope2 | Slope of signal trajectory between first valley and first peak time-points. |
|  | Trunk/pelvis angle flexion | First peak (amplitude, time, prominance and width) | Maximum point of flexion-extension during the stance phase. Value and % of gait cycle when it occurs. |
|  |  | First valley (amplitude, time, prominance and width) | Minimum point of flexion-extension during the stance phase. Value and % of gait cycle when it occurs. |
|  | Trunk/pelvis angle tilt | First peak (amplitude, time, prominance and width) | Maximum point of tilt between initial heel-strike phase and midstance. Value and % of gait cycle when it occurs. |
|  |  | First valley (amplitude, time, prominance and width) | Minimum point of tilt between heel-off and midstance. Value and % of gait cycle when it occurs. |
|  | Trunk/pelvis angle rotation | First peak (amplitude, time, prominance and width) | Maximum point of rotation between midstance and midswing. Value and % of gait cycle when it occurs. |
|  | All inertial sensor signals common features | Initial value | Initial value of signal at time 0. |
|  |  | Flexion-extension distance between maximum and minimum flexion-extension values | Range of motion and distance in % of cycle between maximums and minimums flexion-extension points obtained. |
|  |  | Mean of signal | Mean value of signal during gait cycle. |
|  |  | Variability of signal | Standard deviation of signal during gait cycle. |
|  |  | Area of signal | Area under the curve of flexion-extension during gait cycle. |
|  |  | Signal range of motion | Range of motion of signal during gait cycle. |
|  |  | Signal entropy | Quantifies the uncertainty or randomness in a signal. A higher entropy indicates greater complexity or higher variability, meaning the signal exhibits faster and more unpredictable changes. |
|  |  | Distance and amplitude between peaks and valleys | Distance in % of cycle between maximums and minimums during gait cycle.  Amplitude of the pattern between maximums and minimums during gait cycles. |
|  |  | Peak or valley prominence | Measure that indicates how tall a peak is, compared to the lowest point surrounding it. Reflects the significance of peaks, helping to determine how much it stands out within the signal. |
| Insoles (Moticon) | Vertical force (VF) | First peak (amplitude, time, prominence and width) | Maximum point of flexion-extension between heel-strike and middle-stance phase. Value and % of gait cycle when it occurs. |
|  |  | Second peak (amplitude, time, prominence and width) | Maximum point of flexion-extension between heel-off and toe-off phase. Value and % of gait cycle when it occurs. |
|  |  | First valley (amplitude, time, prominence and width) | Minimum point of flexion-extension between middle-stance and pre-swing phase. Value and % of gait cycle when it occurs. |
|  |  | Slope | Slope of signal trajectory between first valley and second peak time-points. |
|  |  | Range of motion between peaks | Difference of amplitude between first peak and second peak. |
|  | Center of Pressure (COP) | Maximum and minimum value | Maximum point on all axis (X,Y). It determines the range of trajectory of center of pressure during gait cycle. |
|  |  | Slope between maximum and minimum | Slope of trajectory of Center of Pressure during gait cycle. |
|  |  | Average slope change between maximum and minimum | Mean value of derivative gradient during the stance phase of gait cycle. |
|  |  | Regression | Linearity of center of pressure trajectory. |
|  |  | Maximum velocity (derivative of cop trajectory) | Highest speed of center of pressure movement. |
|  |  | Minimum velocity (derivative of cop trajectory) | Lowest speed of center of pressure movement. |
|  |  | Maximum acceleration (derivative of cop velocity) | Peak rate of velocity change. |
|  |  | Minimum acceleration (derivative of cop velocity) | Minimum rate of velocity change. |
|  |  | Root mean square derivative of cop trajectory | Root mean square of velocity change over time. |
|  | All insole signal common features | Distance and amplitude between peaks and valleys | Distance in % of cycle between maximums and minimums during gait cycle.  Amplitude of the pattern between maximums and minimums during gait cycles. |
|  |  | Mean signal | Mean value of signal during gait cycle. |
|  |  | Variability signal | Standard deviation of signal during gait cycle. |
|  |  | Signal range of motion | Range of motion of signal during gait cycle. |
|  |  | Area of signal | Area under the curve of signal during gait cycle. |
|  |  | Signal entropy | Quantifies the uncertainty or randomness in a signal. A higher entropy indicates greater complexity or higher variability, meaning the signal exhibits faster and more unpredictable changes. |
|  |  | Peak or valley prominence | Measure that indicates how tall a peak is, compared to the lowest point surrounding it. Reflects the significance of peaks, helping to determine how much it stands out within the signal. |
|  | Spatiotemporal variables | Distance (m) | Distance covered by the patient during the test. |
|  |  | Number of steps | Steps walked during the test. |
|  |  | Speed (m/s) | Velocity of the patient gait. |
|  |  | Cadence (step/min) | Number of steps per minute. |
|  |  | Stride length (m) | Length in meters of a gait cycle normalized to height. |
|  |  | Stride velocity(m/s) | Velocity in meters / second of gait cycle |
|  |  | Stance phase (%) | Percentage of time in stance phase, foot-ground contact, on the overall gait cycle. |
|  |  | Swing phase (%) | Percentage of time in swing phase, foot in the air, on the overall gait cycle. |
|  |  | Single support (%) | Percentage of time in single support, one foot on the ground, on the overall gait cycle. |
|  |  | Double support (%) | Percentage of time in double support, both feet on the ground, on the overall gait cycle. |
|  |  | Step duration (s) | Time token to perform a step. |
| Surface EMG (Myontec) | Quadriceps EMG signal | First peak (amplitude, time, prominence and width) | Maximum point of activation between initial heel-strike and midstance. Value and % of gait cycle when it occurs. |
|  |  | Second peak (amplitude, time, prominence and width) | Maximum point of activation between initial heel-off and midswing. Value and % of gait cycle when it occurs. |
|  |  | First valley (amplitude, time, prominence and width) | Minimum point of activation between toe-off and the end of swing phase. Value and % of gait cycle when it occurs. |
|  | Hamstrings EMG signal | First peak (amplitude, time, prominence and width) | Maximum point of activation between initial heel-strike and midstance. Value and % of gait cycle when it occurs. |
|  |  | Second peak (amplitude, time, prominence and width) | Maximum point of activation between initial heel-off and midswing. Value and % of gait cycle when it occurs. |
|  |  | Mean signal during stance phase. | Mean value of signal between midstance and final stance phase. |
|  |  | Mean signal during swing phase. | Mean value of signal between toe-off and midswing. |
|  |  | Maximum signal value during swing phase. | Maximum value of signal between toe-off and midswing. |
|  | Gluteus EMG signal | First peak (amplitude, time, prominence and width) | Maximum point of activation between initial heel-strike and midstance. Value and % of gait cycle when it occurs. |
|  |  | Mean signal during stance phase | Mean value of signal between midstance and final stance phase. |
|  |  | Mean signal during swing phase | Mean value of signal between toe-off and midswing. |
|  |  | Maximum signal value during stance phase | Maximum value of signal between midstance and final stance phase. |
|  | All EMG signal common features | Distance and amplitude between peaks and valleys | Distance in % of cycle between maximums and minimums during gait cycle.  Amplitude of the pattern between maximums and minimums during gait cycles. |
|  |  | Mean signal | Mean value of signal during gait cycle. |
|  |  | Variability signal | Standard deviation of signal during gait cycle. |
|  |  | Area of signal | Area under the curve of signal during gait cycle. |
|  |  | Singal entropy | Quantifies the uncertainty or randomness in a signal. A higher entropy indicates greater complexity or higher variability, meaning the signal exhibits faster and more unpredictable changes. |
|  |  | Peak or valley prominence | Measure that indicates how tall a peak is, compared to the lowest point surrounding it. Reflects the significance of peaks, helping to determine how much it stands out within the signal. |
